# The quality of life in papillary thyroid microcarcinoma patients undergoing lobectomy or total thyroidectomy: A cross‐sectional study

**DOI:** 10.1002/cam4.3747

**Published:** 2021-02-26

**Authors:** Yu Lan, Li Cao, Qing Song, Zhuang Jin, Jing Xiao, Lin Yan, Yukun Luo, Mingbo Zhang

**Affiliations:** ^1^ School of Medicine Nankai University Tianjin China; ^2^ Department of Ultrasound General Hospital of Chinese PLA Beijing China; ^3^ Department of Ultrasound The People's Hospital of Liaoning Province Shenyang China; ^4^ Department of General Surgery General Hospital of Chinese PLA Beijing China

**Keywords:** lobectomy, papillary thyroid microcarcinoma, quality of life, surgery, total thyroidectomy

## Abstract

**Objective:**

Papillary thyroid microcarcinoma (PTMC) has a good prognosis and a long survival time, surgery is the common treatment including total thyroidectomy (TT) and unilateral lobectomy (LT), but recent studies showed that TT does not show an advantage over LT for PTMC in preventing cancer recurrence and reducing mortality. Given this, the health‐related quality of life (HRQoL) has become one of the important factors that physicians must consider when making treatment decisions. The aim of this study was to compare the HRQoL of patients between undergoing TT and LT.

**Methods:**

From October 2019 to December 2019, 69 PTMC patients were enrolled in our study, including 34 in the LT group and 35 in the TT group, respectively. We used three questionnaires which included the 36‐item short‐form health survey (SF‐36), thyroid cancer‐specific quality of life (THYCA‐QOL), and Fear of Progression Questionnaire‐Short Form (FoP‐Q‐SF) for each patient to evaluate their scores of HRQoL.

**Results:**

According to the SF‐36, the scores of the domain for the role limitation due to physical problems, emotional problems, and social function (RP, RE, and SF) as well as Physical Component Summary (PCS) and Mental Component Summary (MCS) showed a significant negative linear association between the LT group and TT group: RP (coefficient [coef]: −33.953 [confidence interval (CI) −51.187 to −16.720], *p* < 0.001, RE (coef: −21.633 [CI −39.500 to −3.766], *p* = 0.018), SF (coef: −10.169 [CI −19.586 to −0.752], *p* = 0.035)and PCS (coef: −10.571 [CI −17.768 to −3.373], *p* = 0.005), MCS (coef: −10.694 [CI −19.465 to −1.923], *p* = 0.018). The THYCA‐QOL showed that the scores of the TT group were higher than that of the LT group in the problem of scar (coef: 16.245 [CI 1.697 to 30.794], *p* = 0.029 according to the multivariate analysis), suggesting a higher level of complaint in the TT group. There was no statistically significant difference in the scores of FoP‐Q‐SF between the two groups.

**Conclusions:**

In patients with PTMC, LT offers an advantage over TT in terms of HRQoL, which supports the role of LT as an alternative strategy to TT.

## Graphical abstract

Papillary thyroid microcarcinoma (PTMC) has a good prognosis and a long survival time, surgery is the common treatment including total thyroidectomy (TT) and unilateral lobectomy (LT), but recent studies showed that TT does not show an advantage over LT for PTMC in preventing cancer recurrence and reducing mortality. Given this, the health‐related quality of life (HRQoL) has become one of the important factors that physicians must consider when making treatment decisions. The aim of this study was to compare the HRQoL of patients between under TT and LT.

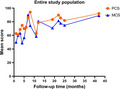

## 1 INTRODUCTION

The incidence of thyroid cancer has been increasing recently,^1^ mainly due to the increased detection of papillary thyroid microcarcinoma (PTMC) as imaging techniques improve. PTMC is considered to be a thyroid papillary carcinoma (PTC) with a maximum diameter of <1 cm,^2^ which has an excellent prognosis and long‐term survival, with a 10‐year survival rate of over 90%.^3^, ^4^ Considering the high morbidity and long survival of PTMC, physicians should pay full attention to the health‐related quality of life (HRQoL) of patients. The latest American Thyroid Association (ATA) guidelines also highlight the significance of incorporating patients' long‐term quality of life into physicians' treatment decision‐making processes.^2^

At present, conventional PTMC treatment is still dominated by surgery, including total thyroidectomy and unilateral lobectomy (with or without cervical lymph node dissection as required).^2^ All patients with total thyroidectomy and a small number of patients with unilateral lobectomy will be required to take the replacement therapy of thyroxine to suppress thyrotropin levels and reduce the risk of recurrence.^5^, ^6^ All of these treatments have potential complications, such as recurrent laryngeal nerve injury, permanent parathyroid dysfunction, or unstable thyroid hormone levels, which may be more likely to occur with total thyroidectomy than with unilateral lobectomy.^7^ The latest ATA guidelines recommend more conservative treatment for low‐risk PTC, such as unilateral lobectomy as an alternative to total thyroid thyroidectomy, and even active surveillance management for PTMC.^2^ The concept is based on the fact that studies have demonstrated that total thyroidectomy does not show an advantage over unilateral lobectomy for PTMC in preventing cancer recurrence and reducing mortality.^8^, ^9^

Despite a good prognosis of PTMC, studies have shown that the quality of life of those PTC patients after surgery is still worse than that of the general population.^10^, ^11^, ^12^ Also, in the ultrasonic diagnosis of daily work, in the face of thyroid cancer patients with a postoperative review, we are surprised to find that the patients underwent total thyroidectomy seems more anxiety and worry than those who underwent unilateral lobectomy, they are very nervous in the entire process of detection. A retrospective study confirmed that the quality of life immediately after unilateral lobectomy was superior to that after total thyroidectomy.^13^ Therefore, we hypothesized that one strategy to reduce anxiety and improve quality of life might be to reduce the extent of surgery. However, at present, there are few comparative studies on the long‐term quality of life patients with different surgical extent.

In this study, we applied three validated questionnaires to explore the differences in the long‐term quality of life of PTMC patients with total thyroidectomy and patients with unilateral lobectomy.

## 2 MATERIALS AND METHODS

### 2.1 Patients

This cross‐sectional study was approved by the ethics review board of our hospital (S2019‐211‐01). The questionnaire process was conducted with the informed consent of each participant. The target population is PTMC patients who underwent thyroid surgery in our hospital from January 2014 to September 2019, including total thyroidectomy and unilateral lobectomy. From October 2019 to December 2019, patients who came to the ultrasound department of our hospital for review and meet the following criteria were enrolled in our study: (1) the classic variant of PTMC confirmed by postoperative pathology; (2) single nodule without imaging evidence of extrathyroidal invasion, lymph node metastasis or distant metastasis; (3) not <1 month's follow‐up. The exclusion criteria are (1) PTMC with aggressive variants (such as tall cell variants, columnar cell variants, hobnail cell variants)^14^; (2) serious chronic diseases (such as respiratory failure, heart failure, kidney failure, etc.) or other cancers (such as breast cancer, liver cancer, cervical cancer, etc.); (3) patients who were illiterate and unable to complete the questionnaire; (4) patients who were clinically diagnosed with mental illness (such as phobias, anxiety, obsessive‐compulsive disorder, etc.).

### 2.2 Surgery process

These included patients were operated on by surgeons (W.T. or Z.Q) with more than 20 years of experience in thyroid cancer surgery in our hospital. These surgeons have more than 1000 cases of thyroid cancer surgery each year. Surgical procedures included total thyroidectomy and unilateral lobectomy with or without cervical lymph node dissection. None of the patients enrolled in our study had been treated with radioactive iodine.

### 2.3 Data collection

#### 2.3.1 Demographic and clinicopathological characteristics

The demographic information was collected in the questionnaire including age, sex, height, weight, level of education, marital status, occupation status, medical expenses, and place to live. The identified co‐morbidities of each participant by physicians were reported and the following disease was registered: diabetes, hypertension, previous stroke, liver failure, kidney failure, chronic pulmonary disease, previous myocardial infarction, atrial fibrillation, rheumatism, depression, or other mental illness, breast cancer, or other cancers. Also, participants were asked to report levothyroxine (LT4) supplementation and the family history of thyroid cancer. The characteristics of the tumor and the surgery options (total thyroidectomy or unilateral lobectomy with or without cervical lymph node dissection) were reviewed from our electronic health records system.

### 2.4 HRQoL questionnaires

#### 2.4.1 Short‐form survey

The Chinese version of SF‐36 as a multipurpose short‐form survey has been well validated and standardized which has been used in many studies to measure HRQoL.^15^, ^16^, ^17^ SF‐36 consists of 36 questions regarding physical and mental wellbeing measuring 8 domains: physical functioning (PF), e.g. “does your health limit your ability to carry groceries?”; role physical (RP), e.g., “is the type of work or activity you want to do limited by your physical health?”; bodily pain (BP), e.g., “have you had bodily pain during the past 4 weeks” general health (GH), e.g., “I think my health is getting worse”; vitality (VT), e.g., “do you find your life fulfilling”; social functioning (SF), e.g. “to what extent has your poor health or mood affected your normal social interaction with your family, friends neighbors, or group over the past 4 weeks?”; role emotional (RE), e.g. “have you been able to accomplish only part of what you wanted to do in the past 4 weeks because of emotional problems” and mental health (MH) e.g. “have you felt bored.” Two total scores can be calculated: physical component summary (PCS) and mental component summary (MCS) representing the physical wellbeing and emotional wellbeing, respectively. Responses on each domain were linearly transformed into scores of 0 to 100 according to the SF‐36 manual. The higher scores indicate better HRQoL.

#### 2.4.2 Thyroid cancer‐specific quality of life questionnaire (THYCA‐QOL)

The Chinese version of THYCA‐QOL was used to assess patients' thyroid‐specific symptoms due to thyroid cancer itself or its treatment complications.^18^ The questionnaire includes 24 questions measuring 7 multiitem domains: neuromuscular, voice, concentration, sympathetic, throat/mouth, psychological, and sensory symptoms, as well as six single domains (problems with the scar, feeling chilly, tingling sensation, weight gain, headaches, and a reduced interest in sex).^19^, ^20^ THYCA‐QOL is provided in two versions with a different recall time frame (4 weeks for the sexuality item and 1 week for the other items), all items were grouped into four levels (1 = “not at all” 2 = “a little” 3 = “quite a bit,” and 4 = “very much”) and were assigned 1–4 scores. The higher scores represent more complaints and poorer HRQoL caused by the thyroid‐specific symptoms.^20^

#### 2.4.3 FoP‐Q‐SF questionnaire scores

This questionnaire was developed by Mehnert et al.,^21^ which has been applied in patients with systemic sclerosis ^22^ and cancer ^23^ with high reliability and validity. It consists of 12 items measuring two domains (Physiological health domain and social family domain). Likert 1–5 score method is adopted. Each item is counted as 1 to 5: “never” to “often.” The scale is self‐rated by patients with a total score of 12–60 points, a higher score indicates a greater level of anxiety about disease progression.

All questionnaires in this study were sent and received by the researcher (Y.L.), who explained the method of filling in the questionnaires. The three questionnaires mentioned above were completed after obtaining the patients' informed consent. And the researcher checked whether the questionnaire was wrongly written or omitted and corrected in time.

### 2.5 Statistical analysis

Categorical variables were expressed as numbers, and continuous variables were presented as the mean and standard deviation or median and quartile. Categorical variables were compared by using the Chi‐square test; continuous variables were compared by using the Mann‐Whitney U test for non‐normally distributed data about HRQoL scores and the Student *t* test for normally distributed data about baseline characteristics. Potential demographic and clinicopathological confounders were identified by using a criterion of *p* < 0.10 to differ between total thyroidectomy group and unilateral lobectomy group.

In the evaluation of HRQoL data, statistical difference alone is deficient, so minimally important (MID) is proposed as a supplement, which is defined as the least important difference.^24^ In the comparison of scores in each domain of SF‐36, the difference of 5–10 points between the two groups was interpreted as a clinically relevant difference, while the difference of 10–20 points and >20 points corresponded to a moderate difference and a considerable difference, respectively.^25^

The age was adjusted in the multivariate model. All p‐values were two‐sided, and *p* < 0.05 was considered as statistically significant difference. The SPSS statistical software (version 24.0; IBM, Inc.) was used to perform all statistical analyses, and the figures were generated using Graph Pad Prism 8.0 (Graph Pad Software, Inc.).

## 3 RESULTS

### 3.1 Baseline characteristics of the patients

**FIGURE 1:**
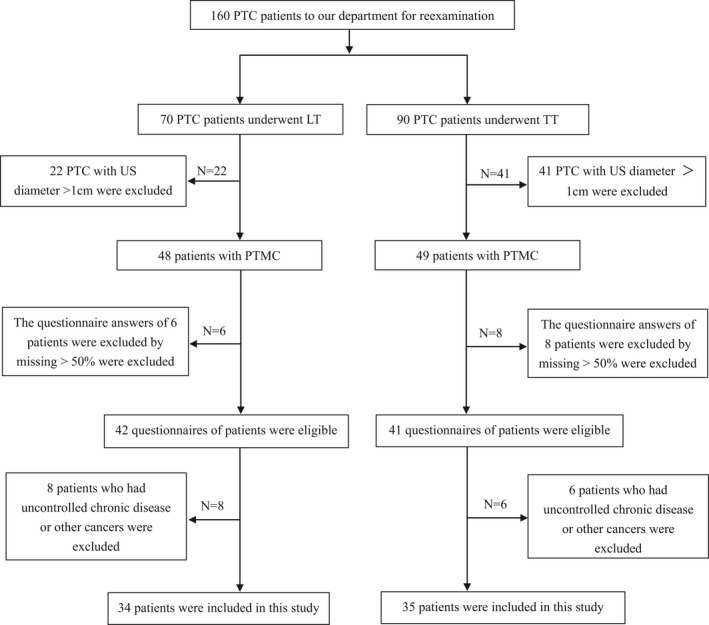
The flow chart of inclusion and exclusion of the lobectomy (LT) and total thyroidectomy (TT) group PTC, papillary thyroid carcinoma; PTMC, papillary thyroid microcarcinoma; US, ultrasound

Sixty‐nine PTMC patients (34 undergoing unilateral lobectomy and 35 undergoing total thyroidectomy) who underwent surgery were enrolled in our study (Figure 1). Baseline characteristics had no difference between the LT group and TT group in age, sex, BMI, marital status, education level, employment status, comorbidity, medical expenses, the place to live, and LT4 supplementation. The proportion of patients followed for more than 24 months was higher in the LT group compared with the TT group (38.2% vs. 20.0%, *p* = 0.095). In addition, in the unilateral lobectomy group, the proportion of patients with a family history of thyroid cancer was slightly higher (14.7% vs. 2.9%, *p* = 0.081) (Table 1).

**TABLE 1:** Baseline characteristics of papillary thyroid microcarcinoma patients in lobectomy (LT) group and total thyroidectomy (TT) group

	Lateral lobectomy (*n* = 34)	Total thyroidectomy (*n* = 35)	*p*‐value
Age (years)	41.44 ± 10.09	43.83 ± 10.71	0.344
Sex			0.198
Male	4 (11.8)	1 (2.9)	
Female	30 (88.2)	34 (97.1)	
BMI	24.47 ± 3.96	24.58 ± 4.06	0.910
Marital status			0.710
Married/partner	30 (88.2)	32 (91.4)	
Living alone	4 (11.8)	3 (8.6)	
Education level			0.280
College degree or higher	16 (47.1)	12 (34.3)	
Others	18 (52.9)	23 (65.7)	
Employment status			0.509
Employed	23 (67.6)	21 (60.0)	
Unemployed	11 (32.4)	14 (40.0)	
Comorbidity			1.000
None	30 (88.2)	30 (85.7)	
Yes	4 (11.8)	5 (14.3)	
Medical expenses			0.947
Public	26 (76.5)	27 (77.1)	
Self‐paying	8 (23.5)	8 (22.9)	
Family history of thyroid cancer			0.081*
No	29 (85.3)	34 (97.1)	
Yes	5 (14.7)	1 (2.9)	
Place to live			0.710
Urban	30 (88.2)	32 (91.4)	
Rural areas	4 (11.8)	3 (8.6)	
LT4 supplementation			0.427
No	0 (0.0)	0 (0.0)	
Yes	34 (100.0)	35 (100.0)	
Follow‐up duration (months)			0.095*
≤24	21 (61.8)	28 (80.0)	
>24	13 (38.2)	7 (20.0)	

Categorical variables were expressed as number and percentage and were compared by using the chi‐square test; continuous variables were presented as the mean and standard deviation and were compared by using the student *t* test.

* *p* < 0.1.

Among the 35 patients who underwent total thyroidectomy, three experienced postoperative voice hoarse. Two patients recovered 1 month after surgery and the other one still not recovered at the time of questionnaire completion (3 months after surgery). No complications occurred in the unilateral lobectomy group in our study.

To find out the factors related to the HRQoL of PTMC patients, univariate analysis was performed. We found that only age is associated with many HRQoL parameters (Table 2). Thus, to control the interference of the confounding factor, the variable for age was adjusted during the multivariate analysis.

**TABLE 2:** Factors related to the quality of life of papillary thyroid microcarcinoma patients

	Age			Sex			family history of thyroid cancer			Follow‐up duration		
	Coef	95% CI	*p*‐value	Coef	95% CI	*p*‐value	Coef	95% CI	*p*‐value	Coef	95% CI	*p*‐value
SF−36												
PCS	−0.245	[−0.519 to−0.154]	0.006**	−6.562	[−21.871 to 8.747]	0.395	0.793	[−13.368 to 14.954]	0.911	1.943	[−6.840 to 10.726]	0.660
PF	−0.406	[−0.773 to −0.039]	0.030**	−6.781	[−21.817 to 8.254]	0.371	−1.508	[−15.420 to 12.404]	0.829	0.056	[−8.587 to 8.700]	0.990
RP	−0.787	[−1.701 to 0.127]	0.090	−5.078	[−42.243 to 32.087]	0.786	5.159	[−29.032 to 39.349]	0.764	15.434	[−5.479 to 36.347]	0.145
BP	−0.672	[−1.017 to−0.327]	0.000**	−13.719	[−28.549 to 1.112]	0.069	−0.758	[−14.745 to 13.229]	0.914	−2.817	[−11.477 to 5.843]	0.518
GH	−0.211	[−0.643 to 0.221]	0.334	−0.669	[−18.001 to 16.663]	0.939	0.278	[−15.670 to 16.225]	0.972	−4.900	[−14.732 to 4.932]	0.323
MCS	−0.186	[−0.623 to 0.251]	0.399	7.032	[−10.380 to 24.444]	0.423	−3.690	[−19.762 to 12.383]	0.648	1.746	[−8.242 to 11.735]	0.728
VT	0.101	[−0.356 to 0.558]	0.660	13.203	[−4.725 to 31.131]	0.146	−4.286	[−21.013 to 12.441]	0.611	−4.219	[−14.577 to 6.138]	0.419
SF	−0.538	[−1.003 to−0.073]	0.024**	−10.417	[−29.487 to 8.654]	0.280	2.469	[−15.222 to 20.160]	0.781	−0.476	[−11.469 to 10.517]	0.931
RE	−0.364	[−1.254 to 0.526]	0.417	21.042	[−14.192 to 56.275]	0.237	−11.640	[−44.277 to 20.996]	0.479	14.762	[−5.263 to 34.787]	0.146
MH	0.057	[−0.353 to 0.466]	0.783	4.300	[−11.992 to 20.592]	0.527	−1.302	[−16.319 to 13.716]	0.863	−3.082	[−12.380 to 6.217]	0.511
THYCA‐QoL												
Neuromuscular	0.197	[−0.190 to 0.584]	0.313	2.535	[−12.993 to 18.062]	0.746	6.349	[−7.864 to 20.563]	0.376	0.601	[−8.278 to 9.479]	0.893
Voice	0.544	[0.025 to 1.062]	0.040**	9.219	[−11.970 to 30.408]	0.388	−10.582	[−30.017 to 8.853]	0.281	−5.000	[−17.115 to 7.115]	0.413
Concentration	0.103	[−0.397 to 0.604]	0.681	−4.635	[−24.555 to 15.284]	0.644	4.101	[−14.230 to 22.431]	0.657	2.534	[−8.850 to 13.918]	0.658
Sympathetic	0.110	[−0.333 to 0.554]	0.621	2.917	[−14.767 to 20.600]	0.743	−9.656	[−25.769 to 6.456]	0.236	−0.289	[−10.402 to 9.824]	0.955
Throat/mouth	0.048	[−0.305 to 0.401]	0.787	−10.313	[−24.170 to 3.545]	0.142	−8.995	[−21.766 to 3.776]	0.164	−0.612	[−8.659 to 7.434]	0.880
Psychological	−0.148	[−0.567 to 0.271]	0.484	−5.859	[−22.563 to 10.844]	0.486	−1.653	[−17.073 to 13.766]	0.831	4.133	[−5.394 to 13.659]	0.390
Sensory	0.093	[−0.387 to 0.574]	0.699	2.708	[−16.440 to 21.857]	0.779	14.286	[−2.996 to 31.567]	0.104	3.503	[−7.412 to 14.419]	0.524
Problems with scar	−0.554	[−1.274 to 0.166]	0.129	13.750	[−15.230 to 42.730]	0.347	14.021	[−12.602 to 40.644]	0.297	−3.878	[−20.521 to12.766]	0.643
Felt chilly	0.332	[−0.439 to 1.104]	0.393	14.896	[−15.782 to 45.573]	0.336	−0.529	[−28.952 to 27.894]	0.970	4.014	[−13.612 to 1.639]	0.651
Tingling hands/feet	0.055	[−0.507 to 0.617]	0.846	16.667	[−5.342 to 38.675]	0.135	7.407	[−13.105 to 27.920]	0.474	−2.993	[−15.761 to 9.775]	0.641
Gained weight	−0.581	[−.03 to 0.041]	0.067	−16.563	[−41.654 to 8.520]	0.192	−8.730	[−32.008 to 14.548]	0.457	2.857	[−11.643 to 17.358]	0.695
Headache	−0.349	[−0.897 to 0.199]	0.208	8.021	[−13.967 to 30.008]	0.469	7.672	[−12.552 to 27.896]	0.452	8.299	[−4.151 to 20.750]	0.188
Less interest in sex	−0.738	[−1.250 to−0.026]	0.005**	−8.854	[−30.367 to 12.658]	0.414	−3.175	[−23.052 to 16.703]	0.751	−2.517	[−14.856 to 9.822]	0.685
FoP‐Q‐SF												
Physical health	−0.003	[−0.023 to 0.017]	0.773	0.033	[−0.772 to 0.838]	0.934	0.368	[−0.367 to 1.103]	0.322	−0.055	[−0.515 to 0.404]	0.811
Social family	−0.007	[−0.027 to 0.013]	0.481	−0.027	[−0.828 to 0.774]	0.946	0.070	[−0.667 to 0.807]	0.850	0.071	[−0.387 to 0.528]	0.759

** *p* < 0.05.

### 3.2 SF‐36 questionnaire scores

**FIGURE 2:**
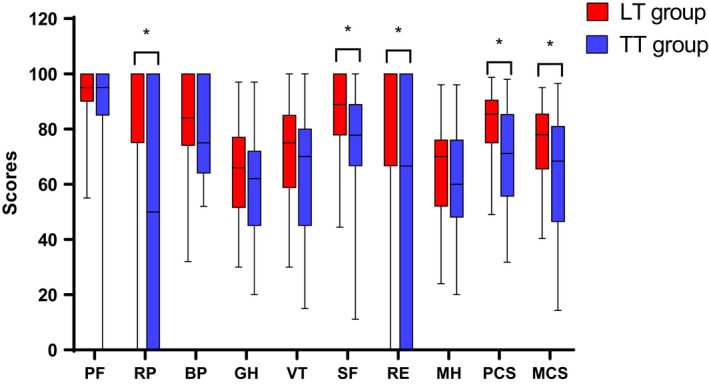
SF‐36 score comparison between patients with papillary thyroid microcarcinoma (PTMC) in the lobectomy (LT) group and in the total thyroidectomy (TT) group: thepatients in the LT group showed significantly higher scores than the TT group in five domains of health‐related quality of life (HRQoL) (*p < 0.05)

The RP, SF, RE, PCS, and MCS scores of patients in the LT group were higher than those in the TT group (Figure 2). The difference of scores in this short survey was 10–20 points in all domains above between the two groups, corresponding to a clinically moderate difference (*p* < 0.05). More importantly, in the univariate and multivariate analyses, the differences in scores were still present and the scores showed a significant negative liner association between the two groups: RP (coefficient [coef]: −33.953 [confidence interval (CI) −51.187 to −16.720], *p* < 0.001, RE (coef: −21.633 [CI −39.500 to −3.766], *p* = 0.018), SF (coef: −10.169 [CI −19.586 to −0.752], *p* = 0.035)and PCS (coef: −10.571 [CI −17.768 to −3.373], *p* = 0.005), and MCS (coef: −10.694 [CI −19.465 to −1.923], *p* = 0.018). The results suggested that both the physical wellbeing and the mental wellbeing of patients in the LT group was better than that in the TT group (Table 3).

**TABLE 3:** Comparison of quality of life in patients with papillary thyroid microcarcinoma underwent lobectomy (LT) versus those who underwent total thyroidectomy (TT)

	Lateral lobectomy (*n* = 34)	Total thyroidectomy^§^ (*n* = 35)	*p*‐value^†^	Univariate analysis			Multivariate analysis		
				Coef	95% CI	*p*‐value	Coef	95% CI	*p*‐value
SF‐36 (the higher score, the better quality of life)									
PCS	85 [75–91]	**71** [56–85]	0.007**	−11.668	[−19.126 to −4.211]	0.003**	−10.571	[−17.768 to −3.373]	0.005**
PF	95 [90–100]	95 [85–100]	0.434	−5.790	[−13.506 to 1.926]	0.139	−4.886	[−12.475 to 2.703]	0.203
RP	100 [75–100]	**50** [0–100]	0.001**	−35.378	[−52.624 to −18.133]	0.000**	−33.953	[−51.187 to −16.720]	0.000**
BP	84 [74–100]	75 [64–100]	0.415	−2.414	[−10.276 to −5.447]	0.542	−0.822	[−8.044 to 6.401]	0.821
GH	66 [52–77]	62 [45–72]	0.442	−3.090	[−12.046 to 5.867]	0.493	−2.622	[−11.654 to 6.411]	0.564
MCS	78 [65–85]	**68** [46–81]	0.037**	−10.995	[−19.662 to −2.327]	0.014**	−10.694	[−19.465 to −1.923]	0.018**
VT	75 [59–85]	70 [45–80]	0.157	−7.458	[−16.727 to 1.811]	0.113	−7.804	[−17.179 to 1.572]	0.101
SF	89 [78–100]	**78** [67–89]	0.037**	−11.317	[−20.904 to −1.729]	0.021**	−10.169	[−19.586 to −0.752]	0.035**
RE	100 [67–100]	**67** [0–100]	0.041**	−22.213	[−39.864 to −4.562]	0.014**	−21.633	[−39.500 to −3.766]	0.018**
MH	70 [52–76]	60 [48–76]	0.430	−2.992	[−11.426 to 5.443]	0.481	−3.169	[−11.719 to 5.380]	0.462
THYCA‐QoL (the lower score, the better quality of life)									
Neuromuscular	11 [8–33]	22 [11–33]	0.578	0.680	[−6.377 to 9.719]	0.680	1.217	[−6.891 to 9.325]	0.765
Voice	0 [0–33]	0 [0–17]	0.553	−1.891	[−12.931 to 9.149]	0.734	−3.232	[−14.064 to 7.601]	0.553
Concentration	0 [0–17]	0 [0–33]	0.410	1.961	[−8.374 to 12.296]	0.706	1.737	[−8.738 to 12.213]	0.742
Sympathetic	17 [0–33]	33 [0–33]	0.271	3.207	[−5.937 to 12.351]	0.486	2.984	[−6.282 to 12.249]	0.522
Throat/mouth	11 [11–25]	11 [11–22]	0.940	−1.849	[−9.138 to 5.441]	0.614	−1.990	[−9.380 to 5.400]	0.593
Psychological	25 [8–33]	33 [17–33]	0.477	1.366	[−7.322 to 10.053]	0.755	1.742	[−7.036 to 10.520]	0.693
Sensory	25 [17–33]	17 [17–33]	0.773	0.700	[−9.234 to 10.634]	0.889	0.484	[−9.585 to 10.553]	0.924
Problems with scar	0 [0–33]	33 [0–33]	0.010**	14.706	[0.009 to 29.403]	0.050	16.245	[1.697 to 30.794]	0.029**
Felt chilly	33 [0–67]	33 [0–33]	0.884	−2.913	[−18.917 to 13.090]	0.717	−3.757	[−19.895 to 12.381]	0.644
Tingling hands/feet	0 [0–33]	0 [0–33]	0.955	−0.448	[−12.053 to 11.157]	0.939	−0.587	[−12.359 to 11.184]	0.921
Gained weight	17 [0–33]	33 [0–33]	0.363	6.050	[−7.041 to 19.142]	0.360	7.539	[−5.362 to 20.439]	0.248
Headache	17 [0–33]	0 [0–33]	0.652	−3.501	[−14.916 to 7.914]	0.542	−2.704	[−14.161 to 8.753]	0.639
Less interest in sex^‡^	33 [25–33]	0 [0–33]	0.038**	−10.392	[−21.314 to 0.529]	0.062	−8.747	[−19.261 to 1.768]	0.101
FoP‐Q‐SF (the lower score, the better quality of life)									
Physical health	2 [2–3]	2 [2–3]	0.555	0.082	[−0.335 to 0.499]	0.695	0.090	[−0.332 to 0.513]	0.671
Social family	2 [1–3]	2 [2–3]	0.477	0.080	[−0.335 to 0.495]	0.702	0.098	[−0.321 to 0.517]	0.641

Data are indicated with medians and quartiles.

† *p*‐value assessed with the Mann–Whitney *U* test.

§ Difference in SF‐36 points printed in bold correspond to moderate clinically difference, i.e. 10–20 points.

** *p* < 0.05;

‡ Higher scores indicate better functioning.

### 3.3 THYCA‐QOL questionnaire scores

**FIGURE 3:**
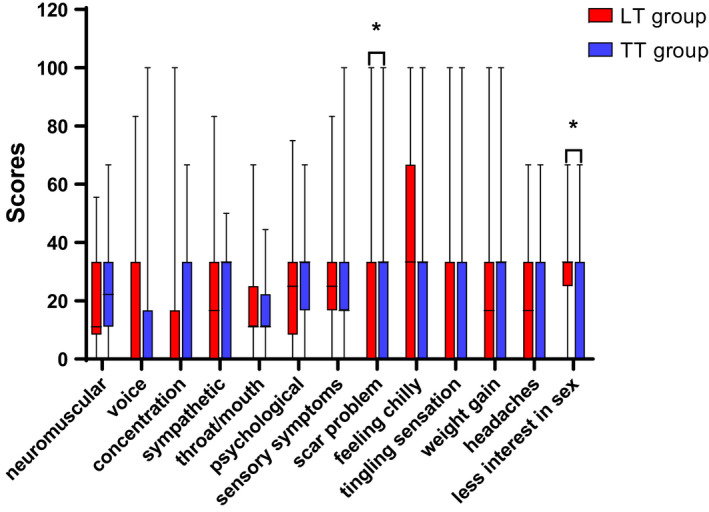
THYCA‐QoL score comparison between patients with papillary thyroid microcarcinoma (PTMC) in the lobectomy (LT) group andin the total thyroidectomy (TT) group: there were statistically significant difference between the two groups in two domains of health‐related quality of life (HRQoL) (*p < 0.05)

The “problems with scar” scale scores of patients in the TT group were higher than those in the LT group, indicating a higher level of the complaint relating to scar symptom (Table 3; Figure 3). Further, the “less interest in sex” scale scores were higher in the patients who underwent unilateral lobectomy than that of those who underwent total thyroidectomy. It indicated that patients in the LT group had better functioning of sex. In both univariate and multivariate analyses, the “problems with scar” scale score showed a significant positive linear association between groups (coef: 16.245 [CI 1.697 to 30.794], *p* = 0.029 according to the multivariate analysis). However, there was no significant difference in the scores of the “less interest in sex” scale between the two groups (*p* = 0.101) (Table 3). It suggested that the difference in sexual interest between the two groups mentioned above may be due to younger patients in the LT group, rather than to the surgical approach.

### 3.4 FoP‐Q‐SF questionnaire scores

Neither physical health nor social family domain had significant differences in FoP‐Q‐SF questionnaire scores between the two groups in all analyses (*p* > 0.05) (Table 3).

### 3.5 The relationship between HRQoL and follow‐up time, age

**FIGURE 4:**
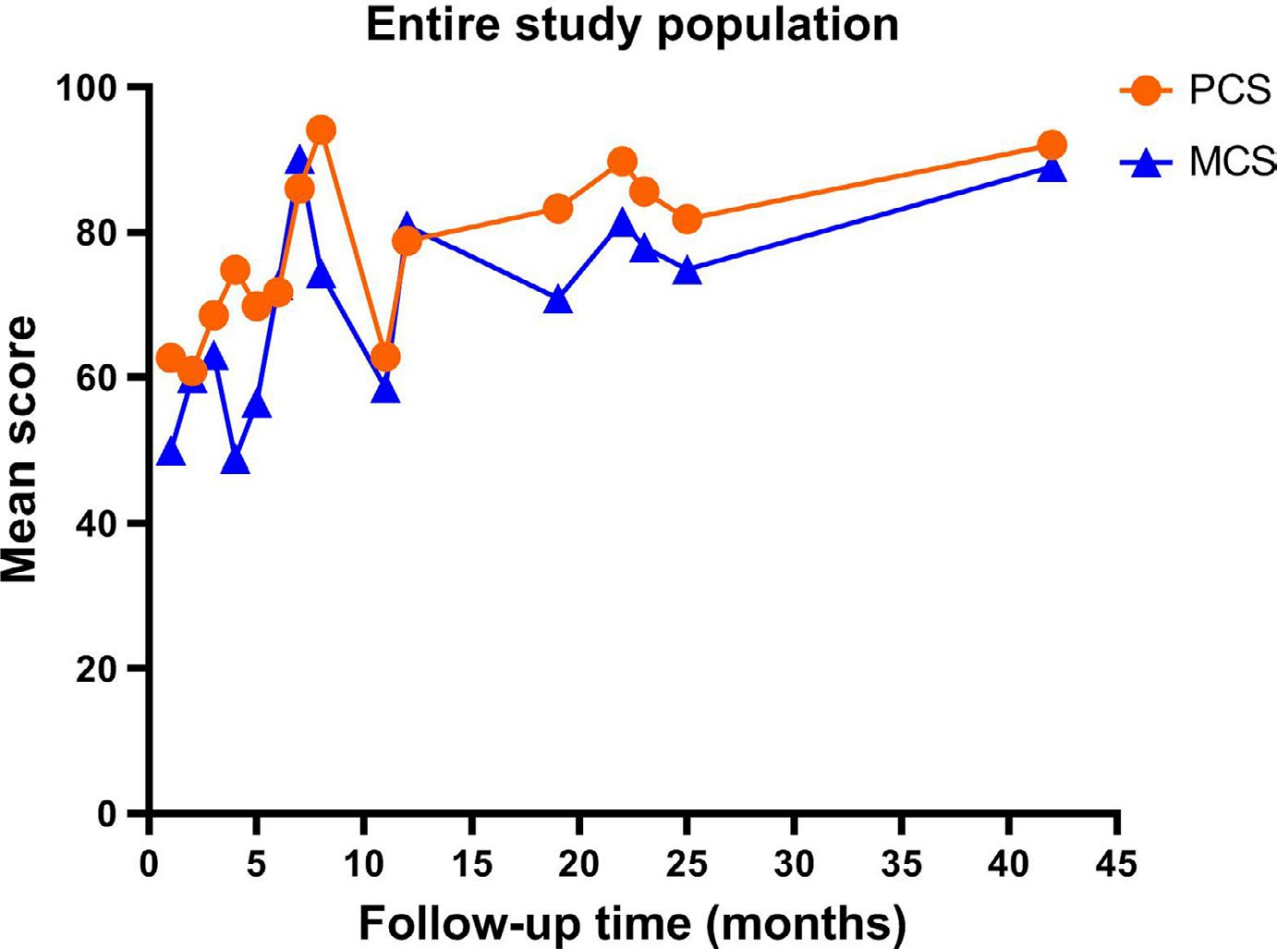
The relationship between health‐related quality of life (HRQoL) and follow‐up time

We found that HRQoL of patients showed a nonliner trend during the 12 months of treatment, as evidenced by several drops in the overall quality of life score including physical wellbeing and mental wellbeing at the early time point. After 12 months, HRQoL plateaus, and then, gradually began to increase over time (Figure 4).

**FIGURE 5:**
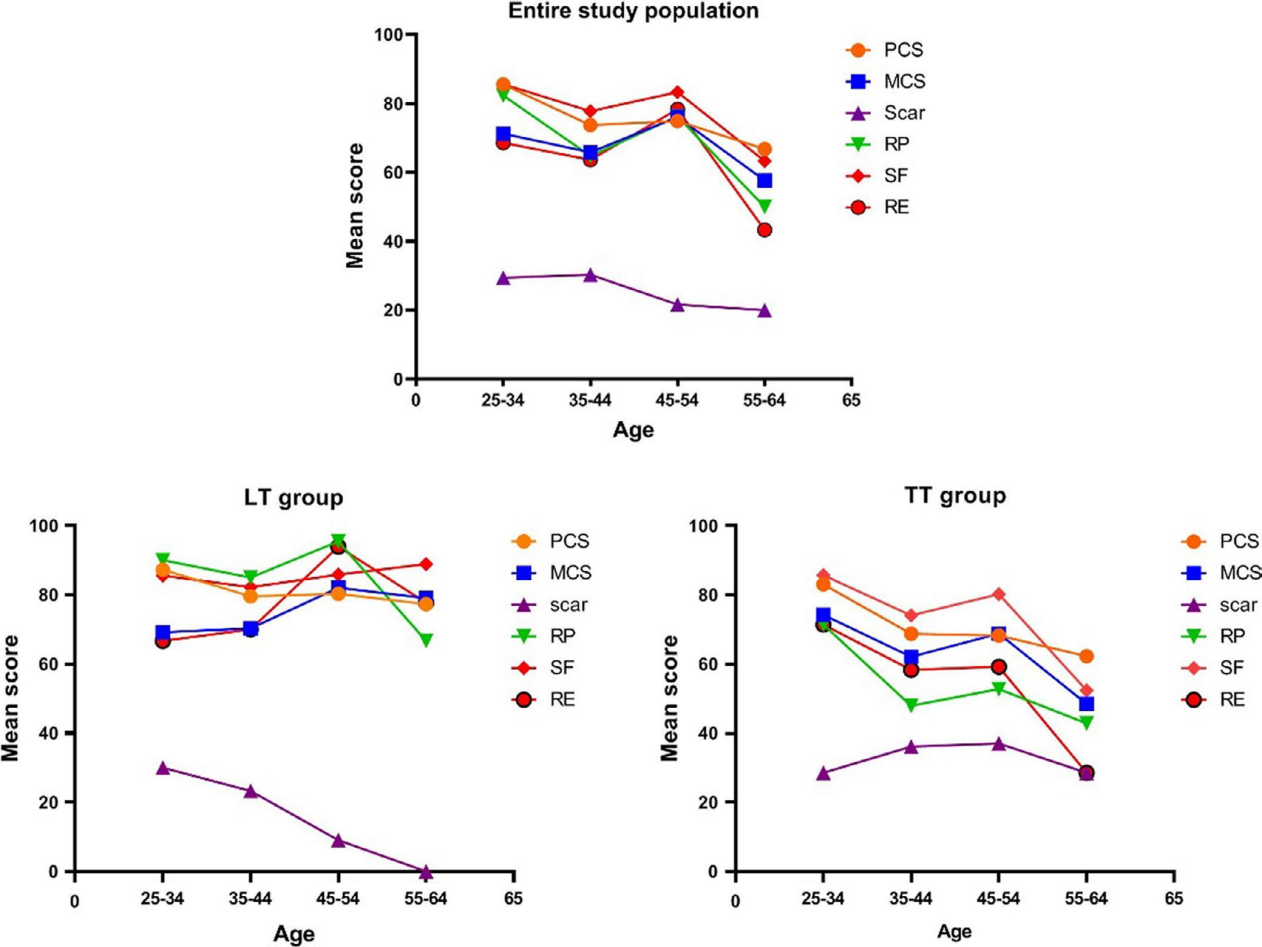
The relationship between health‐related quality of life (HRQoL) and age

In addition, it was found that HRQoL of patients decreased with age, especially in the total thyroidectomy group. In our study, the age group with the best quality of life was 45–55 years old. The age at which quality of life reached its peak was apparent in both the unilateral lobectomy group and the total thyroidectomy group (Figure 5).

## 4 DISCUSSION

The incidence of thyroid cancer is steadily increasing worldwide, especially PTMC.^1^ Although PTMC has an excellent prognosis, studies demonstrated that HRQoL of the survivors is poorer than that of the general population,^26^ and negatively affected for up to 20 years after curative treatment.^27^ Therefore, the goal of PTMC treatment should not only be to prolong survival time, but also to improve the quality of life. HRQoL of thyroid cancer survivors has become an especially significant outcome measure of the treatment.^28^ The latest ATA guidelines also highlight the significance of incorporating patients' long‐term quality of life into physicians' treatment decision‐making processes. In this study, we evaluated the HRQoL of patients with PTMC under different extent of surgery.

Several significant differences were found in HRQoL parameters of the SF‐36 questionnaires between the LT group and the TT group. After adjusting for the confounding factors, the TT group reported more problems associated with PCS, MCS, RP, RE, and SF than the unilateral lobectomy group. RP and RE represent restrictions on daily activities or work due to physical and emotional effects, respectively. SF indicates the ability to take part in social activities. PCS and MCS represent the overall physical and mental health of the patients. The lower scores in these domains in the TT group suggested that the life of the patients who underwent more traumatized was more affected, even their lifestyle and social habits were changed such as paying more attention to diet, deliberately reducing exercise and social activities.

Furthermore, we found that the patients in the TT group reported more scar problems than the LT group. This may be due to the greater scaring of the neck negatively affecting patients' appearance. Quite a lot of patients may think that the apparent scar may have caused damage to their body image, which is a definition of an individual's subjective view of their own body and has to do with self‐esteem and self‐perception, closely related with HRQoL. Sasha K et al. found that there was a statistically significant correlation between neck appearance perception and mental wellbeing parameters such as anxiety, depression, social function, and fatigue in patients.^29^ It suggested that the HRQoL of patients experiencing concern about the scar problem was negatively affected.

Some studies showed that fear of recurrence can negatively affect HRQoL for many years after the treatment of cancer.^30^, ^31^ In our study, the fear of recurrence/metastasis was compared between the two groups. It is encouraged that there was no difference between the two groups in the fear of disease progression especially associated with the fear of second cancer, recurrence, and metastasis. It indicated that patients who chose unilateral lobectomy did not experience a greater fear of progression than those who underwent total thyroidectomy. Similar to some studies in active surveillance (AS) for PTMC patients, the fear of disease progression of the patients in the AS group was similar to that of patients in the surgery group.^32^

In the univariate analysis, we found that age is one of the factors of HRQoL, so we analyzed the parameters of HRQoL in different age groups. The results showed that the HRQoL decreased with age, especially in total thyroidectomy group. This may be due to the fact that older people would have more difficulty with recovery from treatment effects or needed more time to recover, thus, they reported worse outcomes, especially for physical effects. Therefore, for older people, more aggressive treatment strategies should be implemented more conservatively.

The strengths of this study are that it is a comparative study that removed confounding factors, and the goal population is PTMC patients with a relatively high incidence of thyroid cancer. The results can provide a theoretical basis for clinicians to choose a treatment strategy. Furthermore, the three questionnaires used in this study have been demonstrated by previous studies to be validated in evaluating patients' HRQoL. Among them, SF‐36 is considered to be a commonly used and sensitive instrument in measuring HRQoL in thyroid cancer in previous studies.^33^ However, the SF‐36 cannot evaluate all aspects of HRQoL such as disease symptoms or treatment side effects. Thus, the THYCA‐QoL questionnaire and FoP‐Q‐SF were included as a reasonable complement to evaluate important aspects regarding thyroid cancer‐specific symptom ^19^ and fear of the disease progression which may be the strong determinants of the quality of life in thyroid cancer patients.

## 5 STUDY LIMITATIONS

This study has several limitations. First, the number of patients enrolled in our study was limited. Second, the follow‐up time is not long enough, which may overestimate the negative impact of total thyroidectomy on HRQoL,^34^ since the HRQoL of cancer patients may improve over time after surgery.^19^ Last, preoperative quality of life was unknown in both groups, and thus, we cannot evaluate whether the difference of prediagnosis psychological health of patients in the two groups. Thus, prospective studies with large samples and longer term follow‐up are proposed.

## 6 CLINICAL IMPLICATIONS AND CONCLUSIONS

The results of our study suggest that the extent of surgery is one of the important factors affecting the quality of life of thyroid cancer survivors. The recent ATA guidelines recommend unilateral lobectomy as an alternative to total thyroidectomy to minimize the risk of side effects.^2^ From an oncology perspective, data from a large sample showed there was no difference between unilateral lobectomy and total thyroidectomy in cancer recurrence and patients' survival.^35^ From the perspective of global HRQoL of patients, our study suggested that the unilateral lobectomy may have an advantage in improving the HRQoL of patients. Given the results above, PTC patients might suffer from life‐long side effects without obtaining benefit from their treatment.^36^ Thus, less invasive treatment strategies should be considered for PTMC, which supports the role of LT as an alternative strategy to TT. Our study provides an important theoretical basis for clinicians to choose the surgical extent of patients with PTMC.

## 7 ETHICS STATEMENT

The comparative study was approved by the Institutional Review Board at General Hospital of Chinese PLA (S2019‐211‐01).

## CONFLICT OF INTEREST

The authors declare that the research was conducted in the absence of any commercial or financial relationships that could be construed as a potential conflict of interest.

## Data Availability

The data used to support the finds of this study are available from the corresponding author upon request.
